# Effectiveness of hybrid simulation training on medical student performance in whole-task consultation of cardiac patients: The ASSIMILATE EXCELLENCE randomized waitlist-controlled trial

**DOI:** 10.1186/s41077-024-00314-2

**Published:** 2024-10-01

**Authors:** Michael Daly, Claire Mulhall, James O’Neill, Walter Eppich, Jonathan Shpigelman, Caitriona Cahir, Daniel Fraughen, Enda McElduff, Catherine Uhomoibhi, Claire Condron

**Affiliations:** 1 RCSI SIM Centre for Simulation Education and Research, Royal College of Surgeons in Ireland (RCSI) University of Medicine and Health Sciences, 123 St. Stephen’s Green, Dublin, Ireland; 2 School of Medicine, RCSI University of Medicine and Health Sciences, 123 St. Stephen’s Green, Dublin, Ireland; 3 Department of Cardiology, Connolly Hospital, Mill Road, Blanchardstown, Dublin, Ireland; 4 Faculty of Medicine, Dentistry, and Health Sciences, Department of Medical Education and Collaborative Practice Centre, The University of Melbourne, Melbourne, Australia; 5 Data Science Centre, School of Population Health, RCSI University of Medicine and Health Sciences, 123 St Stephen’s Green, Dublin, Ireland

**Keywords:** Hybrid simulation, Whole task, Composite narrative, Cardiology, Clerkship, Consultation

## Abstract

**Background:**

Assessment of comprehensive consultations in medicine, i.e. a complete history, physical examination, and differential diagnosis, is regarded as authentic tests of clinical competence; however, they have been shown to have low reliability and validity due to variability in the real patients used and subjective examiner grading. In the ASSIMILATE EXCELLENCE study, our aim was to assess the effect(s) of expert tuition with hybrid simulation using a simulated patient wearing a novel auscultation vest, i.e. a hybrid simulated patient, and repeated peer grading using scoring checklists on student learning, performance, and acumen in comprehensive consultations of patients with valvular heart disease.

**Methods:**

ASSIMILATE EXCELLENCE was a randomized waitlist-controlled trial with blinded outcome assessment undertaken between February 2021 and November 2021. Students at the Royal College of Surgeons in Ireland in either the second or third year of the four-year graduate-entry medical degree programme were randomized to a hybrid simulation training or waitlist control group and undertook three consultation assessments of three different clinical presentations of valvular heart disease (cases: C1–C3) using hybrid simulation. Our primary outcome was the difference in total score between and within groups across time; a secondary outcome was any change in inter-rater reliability across time. Students self-reported their proficiency and confidence in comprehensive consultations using a pre- and post-study survey.

**Results:**

Included were 68 students (age 27.6 ± 0.1 years; 74% women). Overall, total score was 39.6% (35.6, 44.9) in C1 and increased to 63.6% (56.7, 66.7) in C3 (*P* < .001). On intergroup analysis, a significant difference was observed between groups in C2 only (54.2 ± 7.1% *vs*. 45.6 ± 9.2%; *P* < .001), a finding that was mainly driven by a difference in physical examination score. On intragroup analysis, significant improvement in total score across time between cases was also observed. Intraclass correlation coefficients for each pair of assessors were excellent (0.885–0.996 [0.806, 0.998]) in all cases. Following participation, students’ confidence in comprehensive consultation assessments improved, and they felt more prepared for their future careers.

**Conclusions:**

Hybrid simulation-based training improves competence and confidence in medical students undertaking comprehensive consultation assessment of cardiac patients. In addition, weighted scoring checklists improve grading consistency, learning through peer assessment, and feedback.

Trial registration

ClinicalTrials.gov Identifier: NCT05895799

## Introduction

Comprehensive cardiology consultations are multisensory experiences that require an integration of physical inspection, palpation, and auscultation signs in the context of symptoms and a patient’s history to reach a clinical diagnosis. When performed correctly, accurate cardiac diagnoses can often be made at the bedside, thus enabling more appropriate and expedient diagnostic and therapeutic decisions [1]; however, achieving competence in clinical consultation remains challenging. The integration of clinical data to reach a diagnosis in real time is a highly complex task that is difficult to learn in traditional classrooms. Four fundamental problems in medical education contribute to this complexity: (a) fragmentation — students ineffectively combine elements they learn separately; (b) compartmentalization — learners frequently struggle to integrate their acquired knowledge, skills, and attitudes; (c) low transfer of learning — students often have difficulty applying learnings to new problems and situations; and (d) low levels of realism [2, 3]. Thus, medical students usually learn how to successfully process and integrate real-time clinical information at the bedside of actual patients by observing and/or being taught by experienced and enthusiastic clinician educators [1].

“When used properly, the stethoscope remains a valuable and cost-effective clinical tool that enables many well-trained and experienced clinicians to make rapid and accurate cardiac diagnoses with few, if any, additional investigations required” states Chizner [4]. Nevertheless, competence in cardiac examination skills continue to decline [1, 5–12], and trainees often perform physical examinations inaccurately [5] — an observation with important implications for medical decision-making, patient safety, cost-effective care, and continuing medical education [1, 9, 11, 13, 14]. Undoubtedly, improving the accuracy of students’ physical examination should remain a priority [15] while trying to avoid the aforementioned fragmentation and compartmentalization that may limit transfer of learning.

Simulation-based medical education improves learners’ clinical experience, competence, satisfaction, and self-confidence in individual clinical tasks, e.g. history-taking and physical examination including cardiac auscultation [16]; however, clinical consultation is a complex exercise requiring real-time integration of many individual tasks, clinical skills, processes, insights, and information to facilitate timely decision-making. Whole-task learning models can provide a useful framework to develop learning activities that foster flexibility in complex settings [17, 18]. As such, they can encourage development of efficient problem-solving strategies [18], support complex learning, and foster transfer of learning to the workplace [18–20].

Hybrid simulation typically utilizes wearable or augmented technology in combination with a human actor, i.e. a simulated patient [SP] [21]. In medical education, the technology simulates aspects of a clinical scenario beyond the scope of an SP’s performance, e.g. abnormal clinical signs on cardiac auscultation. As such, hybrid simulation allows delivery of unfragmented clinical scenarios that integrate human interactions with clinical data, thus creating realistic whole-task learning experiences that would be otherwise unachievable in classrooms [21]. Interestingly, the term *hybrid* remains poorly defined in the literature and can cover a wide variety of processes, e.g. the close integration of human actors with technology in the form of a wearable device or the use of a human actor and a high-fidelity simulator, side by side, in the same scenario — but as independent learning modalities that represent the same patient and therefore the whole of the training scenario [21]. Indeed, Lous et al. define hybrid simulation as the use of two or more simulation modalities within the same simulation session [22]. Nevertheless, in a recent systematic literature review of hybrid medical simulations, the authors identified hybrid solutions to a variety of procedures and processes such as tracheostomy, point-of-care ultrasound, intravenous catheter insertion, haemodialysis, and clinical breast examination; however, they failed to identify any published hybrid simulation solution to diagnostic cardiac auscultation in patients with valvular heart disease (VHD) [21]. Hence, the ASSIMILATE EXCELLENCE study (a randomized waitlist-controlled trial) was developed using an instructional design model to assess the effect(s) of hybrid simulation training on the performance of medical students at the Royal College of Surgeons in Ireland (RCSI) Medical School in formative whole-task consultation assessments of cardiology patients with VHD. We hypothesize that medical student performance in these assessments may be enhanced by hybrid simulation training.

## Methods

### Study design

ASSIMILATE EXCELLENCE was a randomized waitlist-controlled study with blinded outcome assessment. At RCSI, medical students in either the second or third year of the four-year graduate-entry medical (GEM) degree programme, who had successfully completed a cardiovascular medicine module, volunteered for inclusion. Students were then randomized into a hybrid simulation training or waitlist control group. All students consented to participate, agreed to assess their assigned peers as peer assessors (PA), and to self-assess their proficiency, satisfaction, and confidence. The data were collected by M. D. and stored on a secure server at RCSI (Dublin). The study was conducted according to the Consolidated Standards of Reporting Trials extension (CONSORT extension) reporting guidelines [23], was approved by the Research Ethics Committee at RCSI (REC202005012), and was registered retrospectively (ClinicalTrials.gov Identifier: NCT05895799) due to the specifics of the included population (students and not patients), which did not require preliminary registration by the Irish authorities.

### Narrative creation, wearable technology design, scoring checklist development, and SP training

**Fig. 1:**
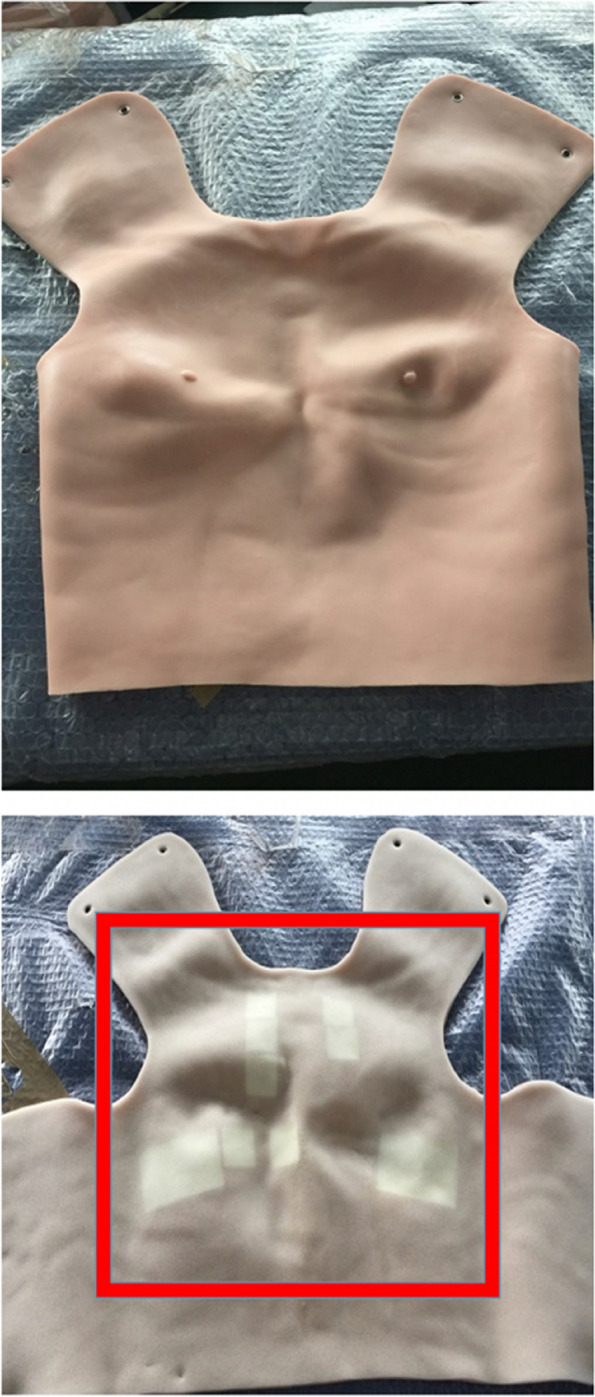
Prototype wearable auscultation vest (*top*, *visible surface*; *bottom*, *underside*) with embedded panel speakers at the anatomically-standardized auscultation positions (*red box*)

Amalgamation of real patients’ stories — or composite narratives — for three different clinical presentations of VHD, typical of those encountered in the final clinical examination of a medical degree programme, were created through iterative consensus by a panel of five clinical experts: (A) paroxysmal nocturnal dyspnoea due to severe mitral regurgitation and atrial fibrillation secondary to mitral valve prolapse in a patient with polycystic kidney disease, (B) exertional syncope due to severe degenerative aortic stenosis in a patient with colonic angiodysplasia, and (C) fever and dyspnoea due to severe aortic regurgitation in a patient with a congenitally bicuspid aortic valve who had recently undergone dental extraction. These composite narratives were then paired to three real patients with the corresponding clinical VHD and auscultation signs; these real patients consented to the recording and storage of their precordial sounds at each anatomically-standardized precordial auscultation positions for use in the trial. Ten prototype wearable skin-like vests with visible surface landmarks and embedded pressure-sensitive panel speakers were adapted to wirelessly transmit these recordings when examined at the anatomically-standardized precordial auscultation positions with a standard stethoscope (Fig. 1). These protypes were tested for functionality pre-study by the research team and faculty assessors (FA). Weighted scoring checklists for the whole-task consultation assessment of each VHD presentation (A–C) were developed (using grading rubrics for summative assessment at RCSI and established reference texts [24, 25]) by the same five clinical experts, i.e. members of the GEM Steering Group at RCSI, responsible for summative assessment design and grading decisions. Ten SPs with extensive experience in medical education were employed; each SP received the scripted narratives one-week prior to each assessment and had a one-hour group pre-briefing session immediately prior to each assessment.

### Randomization and masking

A computer-based software (Research Randomizer 4.0; Social Psychology Network) was used to randomize allocation of the following: (i) the three clinical presentations of VHD (A–C) to each consultation assessment case (case [C] 1–3), (ii) students to either the hybrid simulation training or waitlist control groups, and (iii) two peer performances to each student for grading after each consultation assessment. All assessors were blinded to the students’ training group, weighting of the scoring checklists, and other assessors’ scores in each case.

### Consultation assessments

**Fig. 2:**
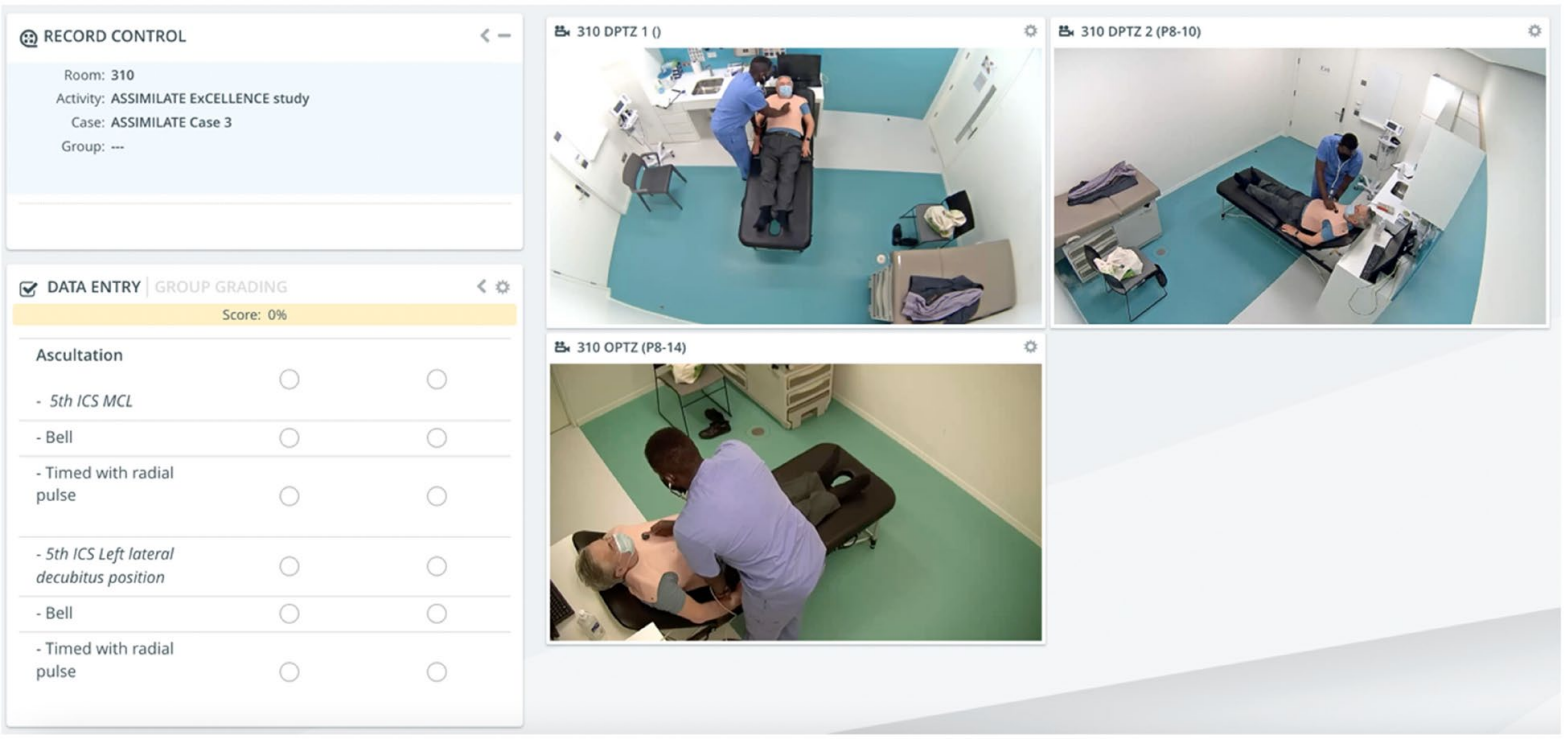
Online grading of the physical examination section in C3 using the CAE LearningSpace Enterprise^™^ online platform

During the study, students participated in three whole-task consultation assessments: (C1) a pre-training assessment to determine baseline competency, (C2) a mid-training programme assessment after randomization and when only those in the hybrid simulation training group had received training from an expert trainer, and (C3) a final assessment six-months after all students had received hybrid simulation training from an expert trainer. Each consultation assessment was performed on an SP wearing our novel auscultation vest and lasted 30 min (15 min for a patient history, 10 min for a physical examination of the cardiovascular system, and 5 min for an oral summary and clinical diagnosis). Audio-visual data for each student performance were recorded and securely stored online for grading (Fig. 2). Ten clinically trained medical educators at RCSI volunteered as FA. To standardize assessment, all FA and PA were trained to use the online grading platform; only FA were provided with the clinical data and scoring checklists for each consultation assessment one-week prior to each grading period. Each aspect of the students’ consultation performance was categorized as either *asked/performed* or *not asked/performed*, with a possible total score of 548 points per consultation assessment (history = 258 points, physical examination = 248 points, diagnosis = 42 points). Each consultation performance was independently graded by two FAs. After each consultation assessment, students were randomly assigned two peer performances to grade using the same online scoring checklist. After each consultation assessment and two-week grading period, students were provided with two FA and two PA scores for their own performance as quantitative feedback. The four total scores for each performance were then assessed for inter-rater reliability [26].

### Hybrid simulation training

Students randomized to the hybrid simulation training group undertook pre-training, i.e. online tutorials on effective history-taking in cardiac patients and physical examination of the cardiovascular system as demonstrated by an expert trainer (each lasting 30 min). Each student then participated in individual in-person hybrid simulation training with an SP wearing our prototype auscultation vest (30 min). Following C2, those students randomized to the waitlist control group received the same online pre-training and individual in-person hybrid simulation training. An expert cardiology trainer facilitated all hybrid simulation training sessions where they provided just-in-time feedback that focused on the processes and accuracy of both clinical performance and VHD diagnoses.

### Students’ self-assessment of proficiency, satisfaction, and confidence

Students anonymously completed an online survey of ten questions (Table 1) before C1 and after C3, ranking responses from 1 (*lowest*) to 10 (*highest*).

**Table 1:** Questions in the online survey

1	How would you rate your history-taking ability in cardiac patients?
2	How would you rate your ability in physical examination of cardiac patients?
3	How would you rate your use of cardiac auscultation in making a diagnosis?
4	How would you rate your ability to combine history-taking and physical examination to reach a diagnosis in cardiac patients?
5	How would you rate your experience(s) in learning cardiac auscultation through simulation?
6	How would you rate your ability to correctly identify abnormal clinical signs using auscultation?
7	How confident are you in your performance in consultation assessments?
8	How confident are you in your performance in cardiology consultation assessments?
9	How anxious are you about your performance in consultation assessments?
10	How prepared do you feel for a career as a practicing doctor?

The survey was iteratively developed by the same five clinical experts on the GEM Steering Group Committee through testing on a random sample of ten RCSI student peers not participating in the study.

### Outcome measures

Our primary end point was the difference in mean FA total score between and within groups across time. Secondary end points were the differences in mean FA section scores, i.e. history, physical examination, and diagnosis scores, between and within groups across time; in addition, inter-rater total score reliability and students’ self-assessment of their proficiency, satisfaction, and confidence across time were also evaluated.

### Statistical analysis

Continuous data are reported as mean ± standard deviation (SD) for normally distributed data or median [interquartile range (IQR)] for non-normally distributed data, as assessed through graphical methods and the Shapiro-Wilk test for normality. Categorical data are reported as counts (percentages). Regarding the primary and secondary end points, data were analysed using a mixed-effects model that included group, case number (C1–3), and their interaction as fixed effects. Given the repeated measures design, the Geisser-Greenhouse correction was applied [27]. Upon finding significant main or interaction effects, post hoc analyses with Bonferroni’s multiple comparisons test and Tukey’s multiple comparisons test were performed for inter- and intragroup comparisons, respectively. Intraclass correlation coefficients (ICC) were calculated to estimate the extent to which assessor pairs conformed when rating the same consultation assessment performance, with a value 0.61–0.80 taken as “Good” and ≥ 0.81 taken as “Excellent” agreement [26]. In analysis of the surveys, the Wilcoxon matched-pairs signed-rank test was used for comparisons of ordinal data. For all analyses, a two-tailed *p* < .05 was considered statistically significant. Statistical analysis was undertaken using Stata/SE 17.0 (StataCorp. 2021, Stata: Release 17, Statistical Software, College Station, TX, USA: StataCorp LLC.) and/or GraphPad Prism version 10.0 (GraphPad Software, Boston, MA, USA).

## Results

### Study population

**Fig. 3:**
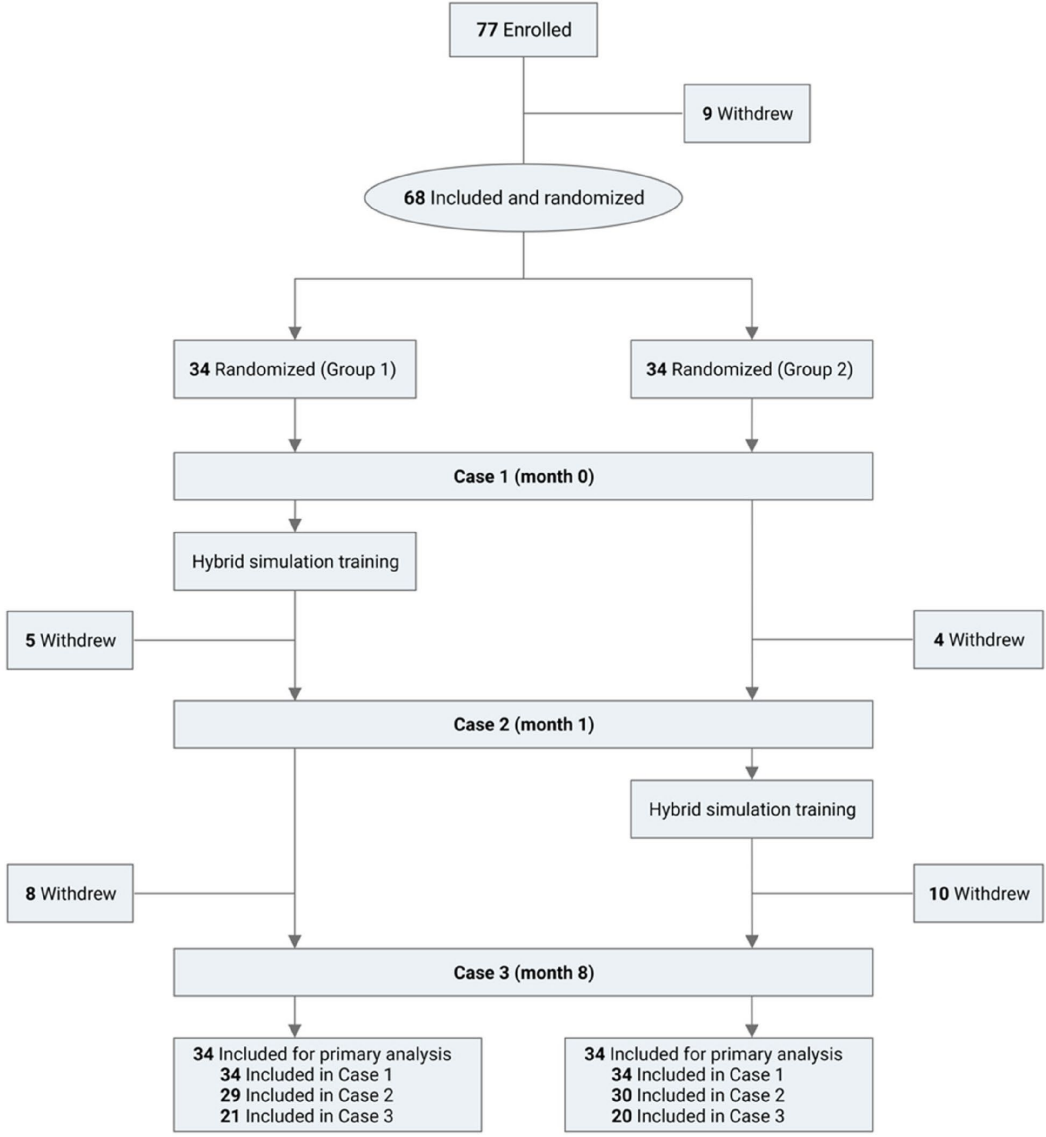
CONSORT flowchart of the study

In February 2021, a total of 77 medical students enrolled in the trial, and 68 were included; of these, 34 were randomized to either the hybrid simulation training (Group 1) or waitlist control group (Group 2). The CONSORT flowchart of the study is shown in Fig. 3. Students who withdrew did so for reasons related to the SARS-CoV-2 pandemic. Baseline characteristics were comparable between groups: overall, participants had a mean age 27.6 ± 0.1 years, were predominantly women (74%) with English as a first language (87%), and were in the second (44%) or third (56%) year of the four-year GEM degree programme at RCSI.

### Primary end point

**Fig. 4:**
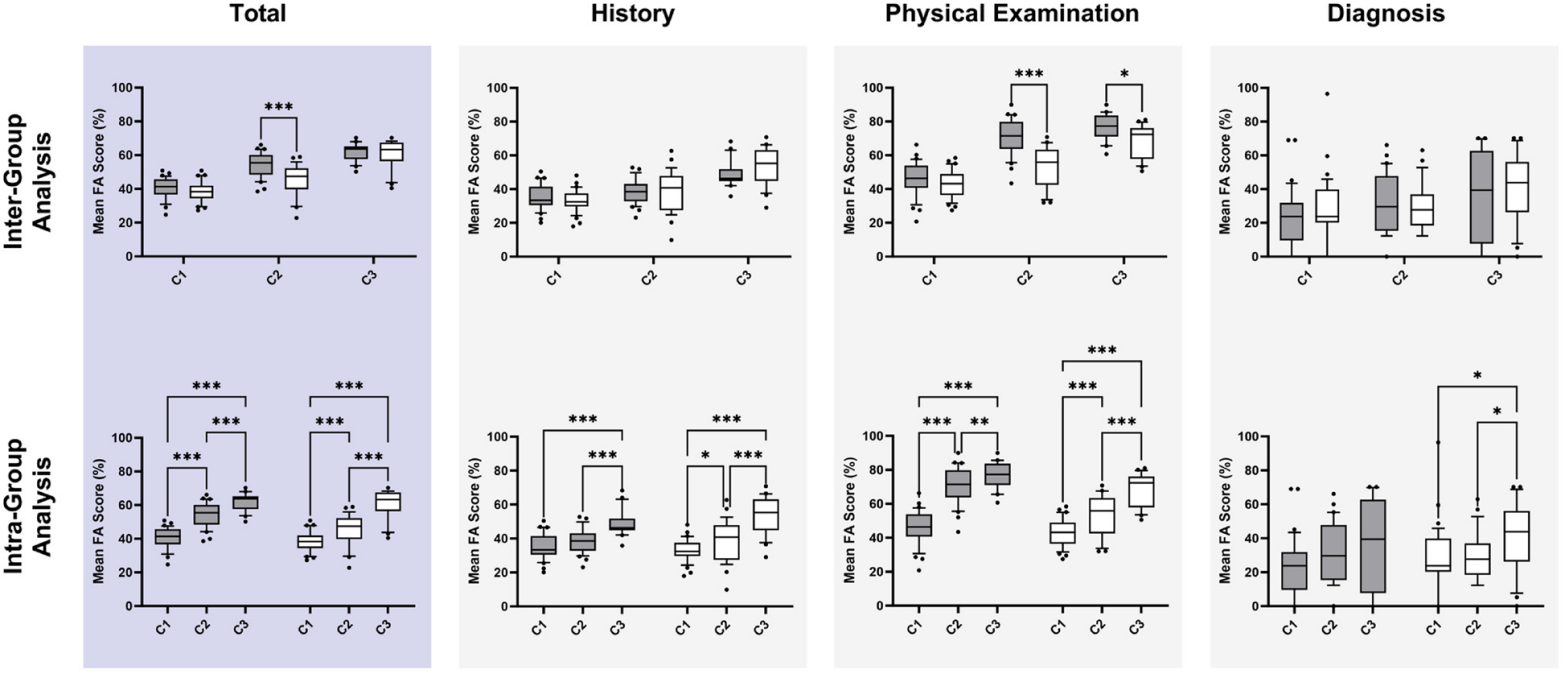
Primary and secondary end points. Box and whisker plots (10–90th percentile) for mean faculty assessor (FA) scores organized by group and case number (C1–C3). Grey boxes represent Group 1; white boxes represent Group 2. Top row shows intergroup comparisons; bottom row shows intragroup comparisons of the same data. Results of the multiple comparisons tests are displayed as pairwise comparisons. *P < .05, **P < .01, ***P < .001

Overall, the median FA total score was 39.6% (35.6, 44.9) in C1 and increased to 63.6% (56.7, 66.7) in C3, i.e. a 60.6% relative increase from baseline (*P* < .001). In the mixed-effects model, all fixed effects were statistically significant: group (F [1.0, 66.0] = 6.7, *P* = .012), case number (F [1.8, 86.5] = 164.4, *P* < .001), and their interaction (F [2.0, 96.0] = 6.7, *P* = .002). On intergroup analysis, a significant difference in mean FA total score was observed in C2 only (54.2 ± 7.1% *vs*. 45.6 ± 9.2%, *P* < .001) (Fig. 4). Within each group, significant improvement in mean FA total score was observed between all cases across time.

### Secondary end points

Results are summarized in Fig. 4 and expressed as percentage scores for each section, i.e. from a possible 258, 248, and 42 points in the history, physical examination, and diagnosis sections, respectively.

### History score

In C1, the median FA score was 33.0% (30.0, 39.2) and increased to 50.0% (44.8, 58.3) in C3, i.e. a 51.5% relative increase from baseline. In the mixed-effects model, the only significant fixed effect was case number (F [1.8, 86.2] = 52.4, *P* < .001); hence, a post hoc analysis was conducted for intragroup analysis only. Within each group, significant improvement in mean FA score was observed between C1 or C2 and C3.

### Physical examination score

In C1, the median FA score was 44.9% (39.8, 51.2) and increased to 75.2% (67.2, 80.0) in C3, i.e. a 67.5% relative increase from baseline. In the mixed-effects model, all fixed effects were statistically significant: group (F [1.0, 66.0] = 22.0, *P* < .001), case number (F [1.9, 90.3] = 170.8, *P* < .001), and their interaction (F [2.0, 96.0] = 14.2, *P* < .001). On intergroup analysis, Group 1 scored significantly higher in C2 (70.9 ± 10.7% *vs*. 52.6 ± 12.2%, *P* < .001) and C3 (77.1 ± 7.7% *vs*. 68.7 ± 9.7%, *P* = .011). Within each group, significant improvement in mean FA score was observed between all cases across time.

### Diagnosis score

In C1, the median FA score was 23.8% (17.0, 34.2) and increased to 43.9% (20.2, 58.8) in C3, i.e. an 84.5% relative increase from baseline. In the mixed-effects model, the only significant fixed effect was case number (F [2.0, 159.7] = 7.3, *P* = .001); hence, a post hoc analysis was conducted for intragroup analysis only. In Group 1, no significant differences were observed; in Group 2, a significant difference was observed between C1 or C2 and C3.

### Inter-rater total score reliability

ICC were calculated for the total scores of each same assessor pair (FA pair or PA pair) in C1–C3. Applying accepted criteria [26], the consistency of the total scores was “Excellent” (0.885–0.996 [0.806, 0.998]) across all cases, implying low variability and high conformity over time.

ICC were also calculated for mixed assessor pairs, i.e. FA/PA, total scores: in C1, the level of agreement was “Good” (0.667–0.686) and improved to “Excellent” (0.850–0.867) in C2 and C3; the lower and upper limits of the 95% CI were 0.461 and 0.921, respectively. Overall, the data were a good fit to the two-way consistency model based on average measurements (*P* < .001). There was no systematic decline in ICC when the sample size was reduced from C1 (*n* = 68) through C2 (*n* = 59) to C3 (*n* = 41), implying that the variation in ICC values was not a simple function of sample size.

### Changes in students’ self-assessment of proficiency, satisfaction, and confidence

**Fig. 5:**
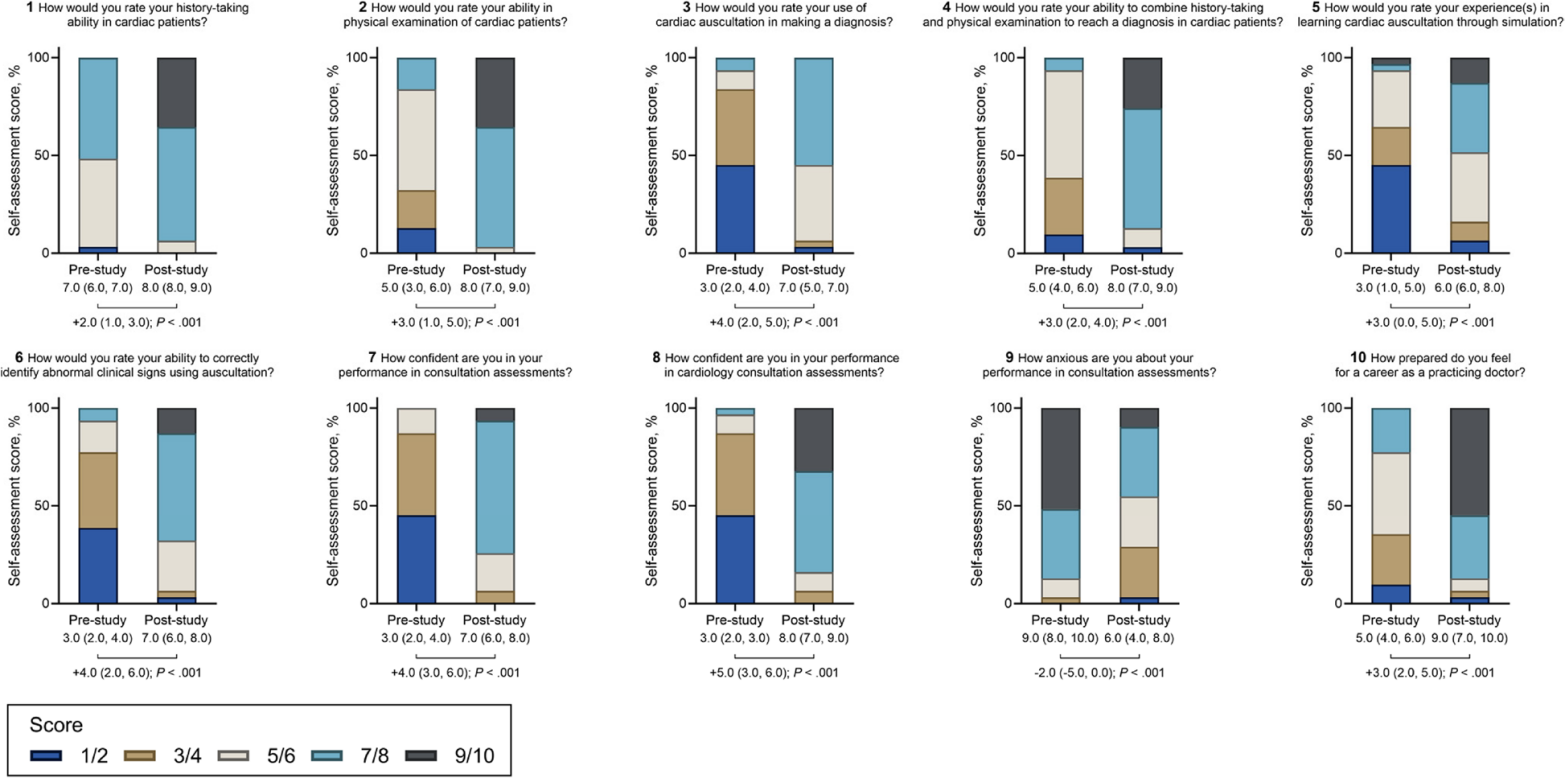
Changes in students’ self-assessment of proficiency, satisfaction, and confidence. The medians (IQR) for the pre- and post-study self-assessment scores and their differences are presented below the corresponding plots

The distributions of responses in the same pre- and post-study surveys for students who completed both (*n* = 31) are summarized in Fig. 5. Following participation, there was significant improvement in how students rated their clinical experience and ability in the various aspects of a comprehensive cardiology consultation (Questions 1–6). In addition, by participating in the study and receiving hybrid simulation training, students’ confidence in their performance in a whole-task consultation assessment improved (Question 7), with a greater improvement reported when asked specifically about cardiac patients (Question 8) (3.0 [2.0, 3.0] *vs.* 8.0 [7.0, 9.0], *P* < .001); perceived anxiety surrounding future consultation assessments (Question 9) also improved (9.0 [8.0, 10.0] *vs.* 6.0 [4.0, 8.0], *P* < .001). Following participation, students felt more prepared for their future careers as practicing physicians (Question 10) (5.0 [4.0, 6.0] *vs.* 9.0 [7.0, 10.0], *P* < .001).

## Discussion

The randomized waitlist-controlled ASSIMILATE EXCELLENCE trial assessed the effectiveness of hybrid simulation training on the whole-task performances of medical students in formative cardiology consultation assessments. Our data demonstrate that hybrid simulation-based training improves performance. Interestingly, all total scores improved across time irrespective of group in our study (Fig. 4). This suggests that participation in hybrid simulated consultation assessments, followed by grading of peer performances with timely faculty and peer quantitative feedback, contributes to learning beyond hybrid simulation training alone. Nevertheless, we observed greater improvement in total score following hybrid simulation training, a finding that was mainly driven by improvements in physical examination score.

Assessments that employ hybrid simulation can allow multiple students to undertake whole-task consultation of the same clinical case under examination conditions [27–30]; in addition, scoring checklists can improve the standardization of both grading and feedback [31]. Thus, the combination of hybrid simulation and scoring checklists could reasonably facilitate direct and quantitative comparison of students’ performances in a whole-task consultation of the same clinical case. In our study, we have successfully simulated whole and real-world doctor-patient consultations of VHD patients through immersive hybrid simulations that can then be scored for clinical and diagnostic accuracy using weighted checklists. Other cardiology simulation models, e.g. Harvey, foster compartmentalized and fragmented skill acquisition [32–35], as students learn on a mannequin with limited vocabulary. The novelty of our approach is that all clinical components of the consultation are necessarily integrated in a realistic and patient-centred manner that assesses diagnostic accuracy in real time and in front of a real human SP with audible cardiac signs. As such, whole-task learning using hybrid simulation models can facilitate realistic and timely integration of skills and processes to address the fragmentation and compartmentalization problems that threaten transfer of learning.

Following participation in our study, the students reported reduced anxiety and improved confidence surrounding future consultation performances, particularly those involving cardiac patients (Fig. 5); in keeping with previous work, satisfaction also improved [36]. Interestingly, Berg and Berg [37] have recently developed a similarly wearable cardiac auscultation vest that plays sounds recorded from real patients; however, they have yet to combine their technology with scripted narratives and to prospectively test its effectiveness in improving learners’ experience and consultation performance.

In essence, the ASSIMILATE ExCELLENCE study prospectively explored the utility of a hybrid simulation-enhanced educational bundle in an international medical student cohort at RCSI. Our unique learning product, i.e. composite narratives in combination with case-specific scoring checklists and a wearable auscultation vest, enables uncompartmentalized whole-task learning through hybrid simulation with potential for transfer of that learning to real patients in the workplace [3]. Importantly, our vest has a realistic skin-like surface with identifiable anatomical landmarks (Fig. 1) that can be used with a traditional stethoscope. In addition, our vest has accurately positioned panel speakers that play complete real-world recordings from actual VHD patients in all auscultation areas and not simply a single area of diagnostic interest, i.e. precordial assessments are authentic, as sounds are heard everywhere and an appreciation of any change(s) in their intensity with a change of auscultation position being a key component of accurate clinical decision-making.

In their recommendations for pre-clerkship clinical skills education, the Association of American Medical Colleges (AAMC) state the following: (a) the primary purpose of clinical skill performance learning is to improve patient outcomes by enhancing the quality of physicians’ care, and (b) pre-clerkship clinical skill education should reflect a patient-centred care strategy and be interactive, experience based, and learner centred [15]. To achieve these outcomes, the ASSIMILATE EXCELLENCE study adopted an instructional design model [38] that was as follows: (a) learner centred and goal oriented and (b) focused on both real-world performance and outcomes that could be reliably measured in both an empirical and valid way [38, 39].

As we know, direct observation of students’ clinical skills in the clerkship years is inconsistent and often limited [40, 41]; consequently, those with performance deficiencies are at risk of ongoing difficulty if they remain unidentified and unremediated [40]. Previous studies have demonstrated that students who underperform on pre-clerkship objective structured clinical examinations (OSCE) also underperform in OSCE later in their medical school curriculum [42–45]. Furthermore, pre-clerkship clinical skills performance aligns with future performances on multiple clerkship outcome measures [40, 46]. In our ASSIMILATE EXCELLENCE study, we have shown that medical student performance is identified and quantified and improves in all domains of the clinical consultation of VHD patients (Fig. 4) through a combination of formative practice, peer grading, timely feedback, and hybrid simulation training. As such, whole-task learning using hybrid simulations of a clinical consultation has the potential to improve students’ performance in high-stakes summative examinations and the quality of their future care of cardiac patients as practicing physicians.

The limitations of our ASSIMILATE EXCELLENCE study were as follows: (a) only GEM students participated over an eight-month period at a single medical school; (b) while 68 students were included, only 60% completed the study due to restrictions of the SARS-CoV-2 pandemic; (c) not all cardiac and/or VHD diagnoses were studied; (d) a combination of echocardiographic data and clinical consensus were used to identify actual patients with both VHD and appropriate clinical signs for use in each case, as there is no gold standard for the accurate identification of clinical signs on physical examination of the cardiovascular system; (e) our vest’s design precluded women as SPs in our study; (f) communication skills and other human factors were not assessed by our weighted scoring checklists; (g) mastery-level scores were not defined in our study; hence, we were unable to compare our scores to those achieved in summative examinations at RCSI; (h) the impact of study participation on students’ performances in final summative clinical examinations at RCSI remain unknown due to ethical constraints; however, permissions should be obtained in any future studies; and (i) longer-term retention of learning and its impact on the quality of real patients’ care remain unknown and are potential areas for future study.

Regarding the evaluation of “retention of learning,” Cepeda et al. suggest that a traditional “spacing experiment” should involve multiple periods of study devoted to the *same* material, separated by a *variable* time gap, with a final *memory test* after a final gap [47]. While ASSIMILATE EXCELLENCE was not designed as a traditional “spacing experiment,” our study did involve multiple periods and types of study, i.e. repeated peer assessment and/or individual teaching from an expert trainer, and multiple formative comprehensive consultation cases (C1–3) separated by variable time gaps — a 30-day gap between C1/C2, a further 30-day gap between C2 and the completion of peer assessment, and then a final 180-day gap between the completion of peer assessment and C3 (Fig. 3). Overall, our results show that total performance scores in formative comprehensive consultations increase linearly between cases that are separated by variable time gaps without any reduction in prior knowledge and/or competence (Fig. 4). As such, we propose that a combination of repeated peer assessment, individual expert teaching, and interval assessment has potential to improve both longitudinal learning, retention, and subsequent performances in whole-task diagnostic clinical consultations.

## Conclusions

This randomized waitlist-controlled trial demonstrates that hybrid simulation-based training results in a significant improvement in the competence and confidence of medical students undertaking whole-task consultation of cardiac patients. Our novel use of SPs wearing an auscultation vest fosters an integrated and comprehensive approach to skills learning and real-time decision-making. In addition, weighted scoring checklists improve grading consistency, learning through peer assessment, and feedback. These results should encourage further investigation into the impact of whole-task learning using hybrid simulation on clinical performance, retention of learning, and the quality of real patients’ care.

## Data Availability

The dataset analysed during the current study is available from the corresponding author on reasonable request.

## Abbreviations

AAMC: Association of American Medical Colleges
C: Case
FA: Faculty assessors
GEM: Graduate-entry medicine
ICC: Intraclass correlation coefficient
IQR: Interquartile range
OSCE: Objective structured clinical examination
PA: Peer assessors
RCSI: Royal College of Surgeons in Ireland
REC: Research Ethics Committee
SD: Standard deviation
SP: Simulated patient
VHD: Valvular heart disease
